# Metastasis Related Epithelial-Mesenchymal Transition Signature Predicts Prognosis and Response to Immunotherapy in Gastric Cancer

**DOI:** 10.3389/fimmu.2022.920512

**Published:** 2022-06-13

**Authors:** Junquan Song, Rongyuan Wei, Shiying Huo, Jianpeng Gao, Xiaowen Liu

**Affiliations:** ^1^ Department of Gastric Surgery, Fudan University Shanghai Cancer Center, Shanghai, China; ^2^ Department of Oncology, Shanghai Medical College of Fudan University, Shanghai, China

**Keywords:** gastric cancer, distant metastasis, epithelial-mesenchymal transition, tumor environment, immunotherapy

## Abstract

**Background:**

Increasing evidence has revealed the effect of epithelial-mesenchymal transition (EMT) on tumor microenvironment and cancer treatment. However, an EMT-based signature to predict the prognosis and therapeutic effect in gastric cancer (GC) has rarely been established.

**Methods:**

Differentially expressed genes (DEGs) between paired primary gastric and ovarian metastatic tumors were identified through comparative RNA-seq analysis, followed by the construction of metastasis-related EMT signature (MEMTS) based on DEGs and EMT gene set. Then, both The Cancer Genome Atlas (TCGA) cohort and the Asian Cancer Research Group (ACRG) cohort were analyzed to explore the potential association between MEMTS and prognosis in GC. Samsung Medical Center (SMC) cohort and two individual immunotherapy treatment cohorts, including Kim cohort and Hugo cohort, were utilized to evaluate the predictive value of MEMTS on the response to adjuvant therapy and immunotherapy, respectively. Finally, the potential association of MEMTS with tumor environment and immune escape mechanisms was investigated.

**Results:**

High MEMTS predicted a poor prognosis in patients with GC. Patients with low MEMTS potentially gained more benefits from adjuvant chemoradiotherapy than those with high MEMTS. MEMTS reliably predicted the response to immunotherapy in GC (area under the curve = 0.896). MEMTS was significantly associated with cancer-associated fibroblasts and stromal score in the aspect of the tumor microenvironment.

**Conclusion:**

MEMTS serves as a potential biomarker to predict the prognosis and response to adjuvant therapy and immunotherapy in GC. MEMTS-based evaluation of individual tumors enables personalized treatment for GC patients in the future.

## Introduction

Gastric cancer (GC) is the fifth most common malignancy and the third leading cause of cancer-related death globally due to its rapid progression and distant metastasis (1). Despite recent advancements in the comprehensive treatment, metastasis remains as a major hindrance to favorable clinical outcomes (2). Recently, multiple therapeutic modes, especially immunotherapy, have become an essential component of treatment and revealed surprisingly powerful effects on protection against tumors (3). However, drug response varies widely even among patients with comparable clinicopathological features (4, 5), implicating those traditional classifications, pathological TNM staging system in particular, are insufficient for the accurate prediction of therapeutic response. Therefore, the development of a novel molecular signature is urgently needed to precisely identify subgroups of GC patients who are more likely to benefit from therapeutic regimens.

Epithelial-mesenchymal transition (EMT) is a common process during embryogenesis, tissue development, wound healing, and carcinogenesis (6). Generally, in the progression of EMT, epithelial cells undergo loss of epithelial polarity, reorganization of their cytoskeleton, and gain of mesenchymal phenotype with more aggressive properties (7). To date, studies have repeatedly uncovered the impact of EMT on tumor microenvironment and tumor treatment. Xu et al. constructed a risk score model based on EMT-related genes (8) whereas Dai et al. established an EMT-related gene signature for predicting clinical outcomes in GC (9). Furthermore, Oh et al. evaluated two distinct molecular subtypes based on an analysis of genomic and proteomic data to identify therapeutic targets and valuable biomarkers for prognosis and therapy response (10). Although these studies revealed the importance of EMT-related scores in GC treatment, they merely evaluated the predictive value of these scores other than immunotherapy and drug resistance. Therefore, it was necessary to establish a reliable and comprehensive evaluation model possessing of better efficacy.

In the present study, we established a metastasis related epithelial-mesenchymal transition signature (MEMTS) and further analyzed the genomic, transcriptomic, and tumor microenvironmental features of different MEMTS subtypes as well as their responses to adjuvant therapy and immunotherapy. We concluded that the MEMTS was a powerful prognostic biomarker and could reliably predict the response to adjuvant therapy and immunotherapy in GC.

## Methods

### Data Sources

Gene expression data in fragments per kilobase million (FPKM) format and corresponding clinical information of gastric cancer in the Cancer Genome Atlas (TCGA) were downloaded from UCSC XENA website (https://xenabrowser.net/datapages/). The FPKM values were converted to transcripts per kilobase millions (TPM) values. The gene expression profiles and corresponding clinical information of Asian Cancer Research Group (ACRG) cohort (GSE66229), SMC cohort (GSE26253), and MD Anderson Cancer Center (MDACC) cohort (GSE28541) were acquired from the Gene Expression Omnibus (GEO) database (https://www.ncbi.nlm.nih.gov/geo/). TCGA cohort and ACRG cohort contained 368 and 300 patients with GC, respectively. In SMC cohort, all patients (n = 432) underwent curative gastrectomy and INT-0116 regimen (5-fluoouracil/leucovorin and radiation) as adjuvant treatment (11). All patients in the MDACC cohort (n = 40) received neoadjuvant chemotherapy or chemoradiation therapy (10). For the microarray data from Affymetrix^®^, the raw “CEL” file was downloaded from GEO database and the microarray data were standardized using the robust multiarray averaging method with the “affy” and “simpleaffy” packages. For the microarray data from other platforms, the normalized matrix files were downloaded directly. The PD-L1 treatment cohorts for gastric cancer (KIM cohort, n = 45) and melanoma (Hugo cohort, n = 26) were obtained from the TIDE database (http://tide.dfci.harvard.edu), which is a computational framework for immunotherapy response prediction. Collectively, TCGA and ACRG cohorts were used to investigate the potential correlation between MEMTS and clinical features and prognosis of GC patients; SMC and MDACC cohorts were used to investigate the predictive role of MEMTS in response to adjuvant therapies; KIM and Hugo cohorts were used to investigate the predictive role of MEMTS in response to immunotherapy. The detailed information of KIM and Hugo cohorts is shown in **Supplementary Tables S1** **-** **2**.

### RNA Sequencing and Identification of the MEMTS Genes

In this study, we obtained the formalin-fixed paraffin-embedded tissues of primary gastric tumors and matching metastatic ovarian lesions of the patients (n = 4) who received gastrectomy without neoadjuvant chemotherapy or radiotherapy between 2016 and 2020 at Fudan University Shanghai Cancer Center. This study was approved by the Ethics Committee of FUSCC and informed consent was received from all patients. For RNA-seq, RNAstormTM FFPE kit (CELLDATA, CA, USA) was used to isolate total RNA. SMARTer Stranded Total RNA-Seq Kit-Pico Input Mammalian Library preparation kit (Clontech, CA, USA) was used to prepare strand-specific RNA-seq library. Qubit fluorometer (Invitrogen, Carlsbad, CA, USA) and Qsep100 (Bioptic, Taiwan, China) were used to check the quality of library. Illumina sequencing platform (Illumina, San Diego, CA, USA) with 150 bp paired-end run metrics was used for performing RNA-seq. The analysis was carried out by “limma” R package and the significance threshold was set as |log2[fold change (FC)] |>1, and False Discovery Rates (FDR) <0.05. The EMT gene set was downloaded from Molecular Signatures Database (http://www.gsea-msigdb.org/gsea/msigdb/). The intersection genes of EMT gene set and upregulated genes in ovarian metastatic tumors were identified as the MEMTS genes, and the average mean of the mRNA expression of MEMTS genes was calculated as MEMTS.

### Survival Prognosis and Genetic Alteration Analysis

The overall survival (OS) and progression-free survival (PFS) for patients were compared by Kaplan-Meier curves with the log-rank test, and the cutoff points were selected by “survival” R package. The somatic mutation data of TCGA-STAD were downloaded from UCSC XENA website. The somatic mutation data were analyzed through R package “maftools”.

### Immune Infiltration Analysis

The immune infiltration among different types of cancers was estimated by CIBERSORT algorithm of “IOBR” R package, which integrated a series of existing algorithms for easy comparison and selection (12, 13). Spearman and distance correlation analysis were used to calculate the correlation of MEMTS and multiple immune cells. Furthermore, the stromal score of each sample was assessed by the R software package “ESTIMATE”, and exclusion score of each sample was calculated in TIDE database (http://tide.dfci.harvard.edu) (14).

### Gene Set Enrichment Analysis

The gene set enrichment analysis (GSEA) method was applied to study the potential mechanisms of MEMTS in the development of gastric cancer. Firstly, we grouped the STAD samples according to the median of MEMTS in all samples, called the “high” group with the MEMTS greater than the median, and the “low” group with less than the median, compared the differences in gene expression between the two groups, and ranked them according to the value of the foldchange. Then we chose the Hallmarker gene sets which were defined based on prior biological knowledge to analyze all samples in the GSEA method using the R package clusterProfiler (version 4.0.5). The normalized enrichment score (NES) was the primary statistic for examining gene set enrichment results. The false discovery rate (FDR) was the estimated probability that a gene set with a given NES represents a false positive finding. We chose NES and FDR as the indicators of enrichment, (Gene sets with |NES|>1 and FDR<0.25 were considered to have significant enrichment) and used the R package “enrichplot” (version 1.12.1) to visualize the results.

### Drug Sensitivity Analysis

The Gene Set Cancer Analysis (GSCA) database (http://bioinfo.life.hust.edu.cn/web/GSCALite/) integrated transcriptome data from the TCGA database, as well as drug response data, enabling comprehensive analysis of gene sets. The “Drug Sensitivity Analysis” module was utilized to analyze the correlation between gene expression and drug sensitivity. Moreover, we predicted each sample’s response to targeted therapy based on the Genomics of Drug Sensitivity in Cancer (GDSC) database through R package “pRRophetic”. Half-maximal inhibitory concentration (IC50) was considered as the indicator of drug sensitivity.

### Statistical Analysis

All statistical calculations were conducted through R software (version 4.1.1). The comparison of differences between two groups was analyzed using Wilcoxon rank sum test. The comparison of differences between three or more groups was analyzed using the one-way ANOVA or Kruskal–Wallis test. The sensitivity and specificity of MEMTS for immunotherapy response prediction were analyzed through the receiver operating characteristic (ROC) curve, and the area under the curve (AUC) was quantified with pROC R package.

## Results

### The Landscape of MEMTS in Gastric Cancer

**Figure 1:**
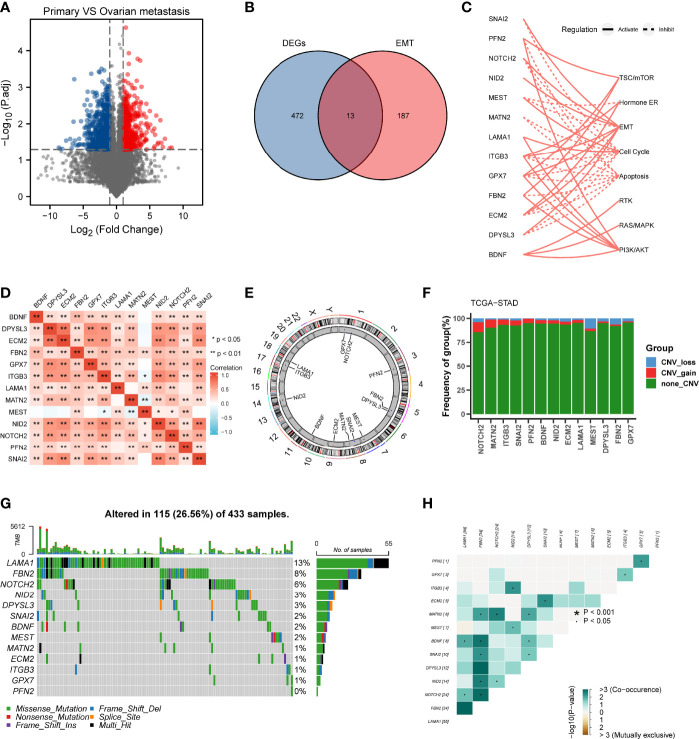
Landscape of genetic variation and correlation of MEMTS in gastric cancer. **(A)** Volcano plot showing DEGs between primary gastric tumor and ovarian metastatic tumor. **(B)** Venn diagram showing 13 genes extracted through taking the intersection of DEGs and EMT hallmark genes. **(C)** The relationships between 13 genes and pathways. The solid and dashed lines denote regulation of activation and inhibition, respectively. **(D)** Spearman’s correlation analyzing the correlation among 13 genes. Orange represents positive correlation and the depth of the color represents the size of correlation coefficient. **(E)** The distribution of 13 genes in the chromosomes. **(F)** CNV analysis of hub genes in TCGA cohort. **(G)** Oncoplots showing the mutation landscape of hub genes in GC patients from TCGA database. Each column represents an individual sample. The upper and right barplots denote TMB and the proportion of each variant type respectively while the mutation frequency in each gene is displayed by the numbers on the right. **(H)** The correlation among mutation of 13 genes. Green represents co-occurrence of mutations in two genes.

The differential gene expression analysis was conducted between primary gastric tumor and ovarian metastatic tumor in FUSCC cohort **(** **Figure 1A** **)**. In view of the significant relationship between tumor metastasis and EMT, we examined the intersection of EMT gene set and DEGs upregulated in ovarian metastatic tumor. Thirteen genes (SNAI2, PFN2, NOTCH2, NID2, MEST, MATN2, LAMA1, ITGB3, GPX7, FBN2, ECM2, DPYSL3, BDNF) were identified as hub genes and subsequent analyses focused on them **(** **Figure 1B** **)**. Functional investigation of hub genes revealed that they could activate TSC/mTOR pathway, ER hormone, EMT, RTK pathway, RAS/MAPK pathway, and PI3K/AKT pathway but inhibit cell cycle and apoptosis **(** **Figure 1C** **)**, which indicated the predominant role of our hub genes in cancer progression and metastasis. Spearman correlation analysis between hub genes showed that most hub genes were significantly correlated with others **(** **Figure 1D** **)**. We further analyzed the distribution of hub genes in the chromosomes and showed that all of them were located in the autosomes **(** **Figure 1E** **)**. Considering the critical role of copy number variation and gene mutation in cancer progression (15), we conducted CNV analysis in the TCGA cohort and demonstrated that NOTCH2, MATN2, and MEST had the higher frequency of CNV among the 13 hub genes **(** **Figure 1F** **)**. On the other hand, we analyzed the mutation landscape of hub genes in GC patients, which showed that LAMA1 (13%) had the highest mutation frequency, followed by FBN2 (8%), NOTCH2 (6%), NID2 (3%), and DPYSL3 (3%), whereas the lowest mutation frequency was possessed by PFN2 (0%) **(** **Figure 1G** **)**. Of note, LAMA1, FBN2, NOTCH2, NID2, and DPYSL3 with a higher mutation frequency were significantly co-occurrent with other genes (**Figure 1H**).

### Characterization of Molecular Features of MEMTS-High and -Low Subtypes

**Figure 2:**
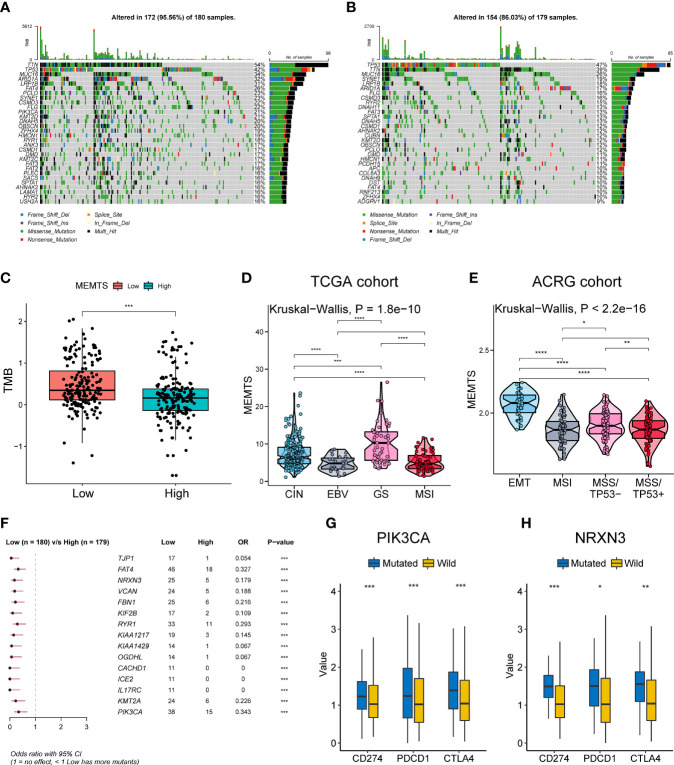
Relationship among the MEMTS, genomic alterations, and molecular subtypes in gastric cancer. **(A, B)** Oncoplots showing landscapes of genomic alterations in low and high MEMTS subtypes, respectively. **(C)** Differences in tumor mutation burden between low and high MEMTS subtypes (***P < 0.001, Wilcoxon test). **(D, E)** The correlations between the MEMTS and TCGA molecular subtypes (Kruskal-Wallis, P = 1.8 × 10^-10^), as well as ACRG molecular subtypes of gastric cancer (Kruskal-Wallis, P < 2.2 × 10^-16^). The plot shows the median value, the 25^th^, and 75th percentiles (central lines, bottom, and top of the box), while the whiskers embody 1.5 times the interquartile range (*P < 0.05; **P < 0.01; ***P < 0.001, ****P < 0.0001, Wilcoxon test). **(F)** Top 15 genes with the highest mutation frequency related to the MEMTS in the TCGA cohort. **(G, H)** PIK3CA and NRXN3 mutations distinctly facilitated expression of immune checkpoints (CD274, PDCD1, CTLA4) (*P < 0.05; **P < 0.01; ***P < 0.001, Wilcoxon test).

We further analyzed genomic alterations in MEMTS-high and MEMT-low subtypes. There was a rough similarity in the kinds of the top 30 genes with the highest mutation frequency between the low and high MEMTS subtypes, while mutation frequency of each gene in the MEMTS-low subtype was almost higher than that in the MEMTS-high subtype (**Figures 2A, B**). Tumor mutation burden has been emerging as a potential immunotherapy biomarker due to the generation of immunogenic neoantigens (16). Hence, we analyzed the differences in tumor mutation burden in two subtypes, and the result showed low MEMTS subtype had a higher level of tumor mutation burden compared to MEMTS-high subtype **(** **Figure 2C** **)**.

Based on different molecular characteristics, gastric cancer was classified into four molecular subtypes respectively in TCGA and ACRG cohort. We found that the MEMTS was the lowest in the Epstein–Barr virus (EBV) subtype and microsatellite instability (MSI) subtypes in the TCGA cohort **(** **Figure 2D** **)**. In the ACRG cohort, we found that molecular subtype in possession of the highest MEMTS was the epithelial-mesenchymal transition (EMT) subtype, while that in possession of the lowest MEMTS was the microsatellite instability (MSI) subtype **(** **Figure 2E** **)**. Then we singled out the top 15 mutated genes which positively related to the MEMTS in the TCGA cohort **(** **Figure 2F** **)**. Considering that high expression of immune checkpoints indicated better response to immunotherapy, we investigated the correlation between these mutated genes and the expression of immune checkpoints. The result showed that the expression of immune checkpoints (CD274, PDCD1, and CTLA4) in patients with PIK3CA or NRXN3 mutation was significantly higher than those in patients with PIK3CA or NRXN3 wild type **(** **Figures 2G, H** **)**.

### The Correlation Between MEMTS and Clinical Features and Prognosis

**Figure 3:**
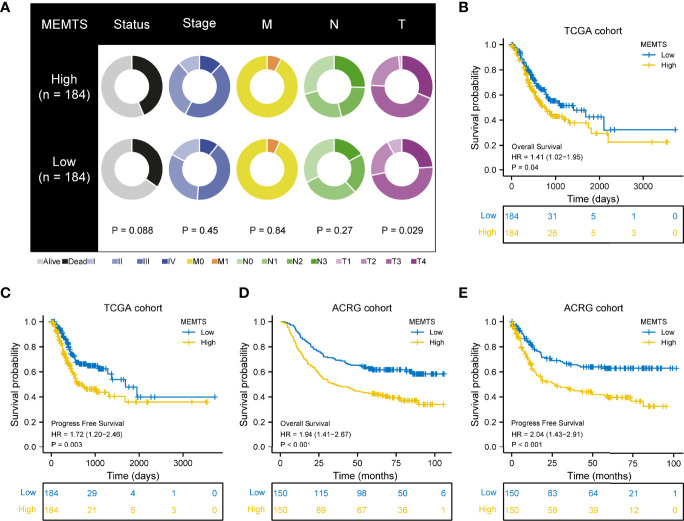
Correlation between MEMTS subtypes and clinicopathological features and prognosis in TCGA and ACRG cohort. **(A)** Different clinicopathological features of high and low MEMTS subtypes in TCGA cohort. **(B–E)** Kaplan–Meier survival plots showing the differences of overall survival and progression-free survival between high and low MEMTS.

Next, we compared the clinical features in MEMTS-low and MEMTS-high subtypes in both TCGA and ARCG cohorts. We demonstrated that the proportion of patients at T4 stage was higher in MEMTS-high subtype whereas the proportion of patients at T1 stage was higher in MEMTS-low subtype **(** **Figure 3A** **)**. In terms of clinical outcome, the prognosis of patients with high MEMTS was poor when compared to patients with low MEMTS in terms of both progression-free survival (PFS) and overall survival (OS) **(** **Figures 3B–E** **)**, which indicated that MEMTS could predict prognosis in patients with GC.

### MEMTS Predicted the Response to Adjuvant and Targeted Therapy in GC

**Figure 4:**
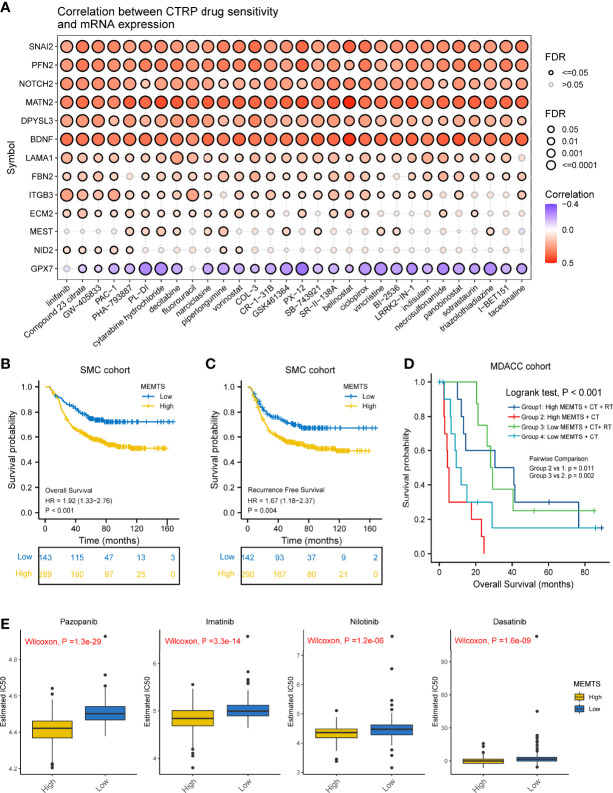
Prediction and correlation of the sensitivity to chemotherapy drugs in gastric cancer. **(A)** The correlation between GDSC drug sensitivity and hub genes expression. **(B)** Kaplan–Meier survival plots showing the differences of overall survival and recurrence free survival between high and low MEMTS. **(C)** Postoperative chemoradiotherapy can significantly improve the prognosis of patients with high and low IMS compared to chemotherapy. **(D)** Kaplan–Meier plot showing the OS of low and high subgroup stratified by MEMTS in patients treated with neoadjuvant chemotherapy or neoadjuvant chemoradiotherapy (MDACC cohort). **(E)** The high MEMTS subtype was related to the lower IC50 of multiple targeted drugs including pazopanib, imatinib, nilotinib, and dasatinib.

It has been demonstrated in various clinical research that adjuvant chemotherapy could improve the prognosis of patients with advanced gastric cancer compared to surgery alone (5, 17). However, drug resistance caused by EMT remained a large obstacle to chemotherapy response. We also investigated the correlation between drug sensitivity and hub genes expression based on the Cancer Therapeutics Response Portal (CTRP) database. The results showed the expression of most of the hub genes was positively related to the IC50 of cancer therapy drugs while the expression of GPX7 was the opposite **(** **Figure 4A** **)**. These results could be used to guide the formulation of chemotherapy regimens.

Then, we investigated the correlation between MEMTS and the response to adjuvant chemoradiotherapy in 432 patients who received homogeneous chemoradiotherapy (5-fluoouracil/leucovorin combined with radiation) after surgery from the SMC cohort. The results showed that patients with low MEMTS gained more benefits in both overall survival and recurrence-free survival than patients with high MEMTS **(** **Figures 4B, C** **)**. Analysis aimed at the MDACC cohort showed that the high MEMTS subtype benefited more from neoadjuvant chemoradiotherapy than neoadjuvant chemotherapy **(** **Figure 4D** **),** which suggests that neoadjuvant chemoradiotherapy was suitable for patients with high MEMTS. However, the result need to be treated with caution due to the limited number of patients. Interestingly, drug sensitivity analysis uncovered that the IC50 of multiple targeted drugs such as pazopanib, imatinib, nilotinib, and dasatinib in MEMTS-high subtype was significantly lower than those in MEMTS-low subtype **(** **Figure 4E** **)**, which indicates that patients with high MEMTS are probably more sensitive to targeted therapy. To sum up, MEMTS was instrumental in predicting the response to adjuvant therapy for GC patients. The patients with low MEMTS subtype might benefit from adjuvant chemotherapy while the patients with high MEMTS subtype might benefit from targeted therapy.

### MEMTS Predicted the Response to Immunotherapy in GC

**Figure 5:**
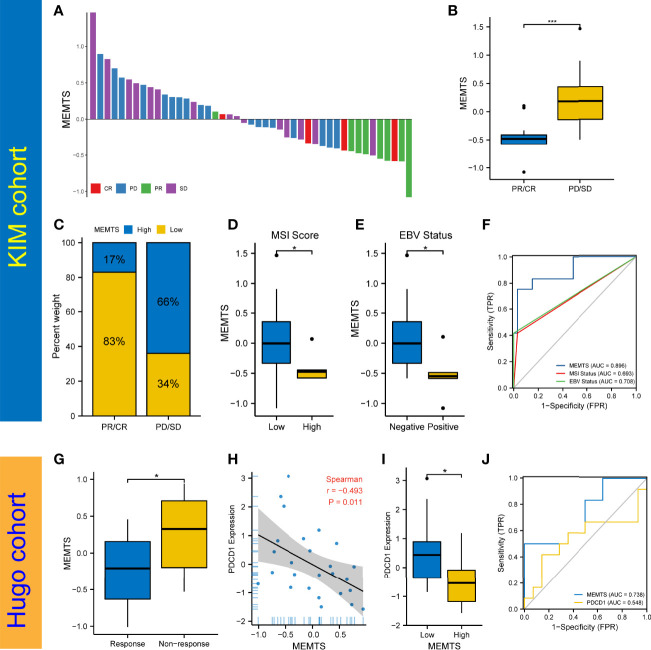
Prediction of the response to immune checkpoint blockade treatment. **(A)** The correlation of MEMTS with response to immunotherapy in KIM cohort. CR, complete response; PD, progressive disease; PR, partial response; SD, stable disease. **(B)** Difference in MEMTS between PR/CR group and PD/SD group (***P < 0.001, Wilcoxon test). **(C)** The proportion of patients with different response to immunotherapy in two MEMTS subtypes. **(D)** Difference in MEMTS between low group and high MSI score group (*P < 0.05, Wilcoxon test). **(E)** Difference in MEMTS between negative and positive EBV status (*P < 0.05, Wilcoxon test). **(F)** The predictive value of MEMTS, MSI status and EBV status in patients treated with immunotherapy (AUC of MEMTS, 0.896; AUC of MSI status, 0.693; AUC of EBV status, 0.708). **(G)** boxplot showing patients with no response to immunotherapy had the higher MEMTS than patients with response to immunotherapy (*P < 0.05, Wilcoxon test). **(H)** Spearman analysis of correlation between MEMTS and PDCD1 expression in Hugo cohort (r = -0.493, P = 0.011). **(I)** Difference in PDCD1 expression between low and high MEMTS subtypes in Hugo cohort (*P < 0.05, Wilcoxon test). **(J)** The predictive value of MEMTS, PDCD1 in Hugo cohort (AUC of MEMTS, 0.738; AUC of PDCD1, 0.548).

The advent of immunotherapy typically represented by PD1/PDL1 checkpoint inhibitors served as an important milestone in tumor treatment. Nivolumab, a monoclonal PD-1 blockade, has recently been approved as first-line treatment in patients with advanced or metastatic gastric cancer in America (18). Considering the promising efficacy of immunotherapy, especially inhibitors of immune checkpoints such as PD-1 and PD-L1, in multiple malignancies including GC, we further evaluated the predictive role of MEMTS in KIM cohort and Hugo cohort. On one hand, we assessed the relationship between MEMTS and immunotherapy responses in KIM cohort, which was made up of advanced GC patients receiving PD-L1 blockade treatment. The MEMTS of patients with different therapeutic effect are shown in **Figure 5A**. The MEMTS of patients in the progressive disease (PD)/stable disease (SD) group was significantly higher than that in the partial response (PR)/complete response (CR) group **(** **Figure 5B** **)**. Notably, MEMTS-low was the dominant subtype (83%) in the PR/CR group while MEMTS-high was dominant subtype (66%) in the PD/SD group, suggesting that MEMTS was an unfavorable factor for immunotherapy response in GC **(** **Figure 5C** **)**. We found that MEMTS was negatively correlated with expression of PD-L1 and PD-1 **(** **Supplementary Figure S1** **)**. Of note, the expression of PD-L1 and PD-1 was reportedly correlated with the response to immunotherapy and clinical outcomes in both major laboratory studies and clinical trials such as Checkmate-649 (19, 20). Furthermore, Both MSI score and EBV status are reportedly the major indicators for the efficacy of immunotherapy (18, 21). Compared with patients with high MSI score or positive EBV status, patients with low MSI score or negative EBV status possessed higher MEMTS **(** **Figures 5D, E** **)**. Correspondingly, we established the AUC of MEMTS and revealed that the AUC value of MEMTS (0.896) was higher than that of MSI score (0.693) and EBV status (0.708) **(** **Figure 5F** **)**. On the other hand, we conducted similar analyses in the Hugo cohort consisting of melanoma patients treated with PD1 blockade. Likewise, patients in the response group had lower MEMTS compared to those in the non-response group **(** **Figure 5G** **)**. In addition, MEMTS was negatively correlated with expression of PDCD1 **(** **Figures 5H, I** **)**, the protein-coding gene of PD-1, whereas the AUC value of MEMTS was higher than that of PDCD1 expression **(** **Figure 5J** **)**, indicating the potential predictive value of MEMTS in terms of immunotherapy response in patients with multiple malignancies. In summary, as patients of MEMTS-low subtype were more likely to benefit from immunotherapy and vice versa, MEMTS could serve as a promising prediction index of immunotherapy response in GC patients.

### MEMTS and Tumor Microenvironment in GC

**Figure 6:**
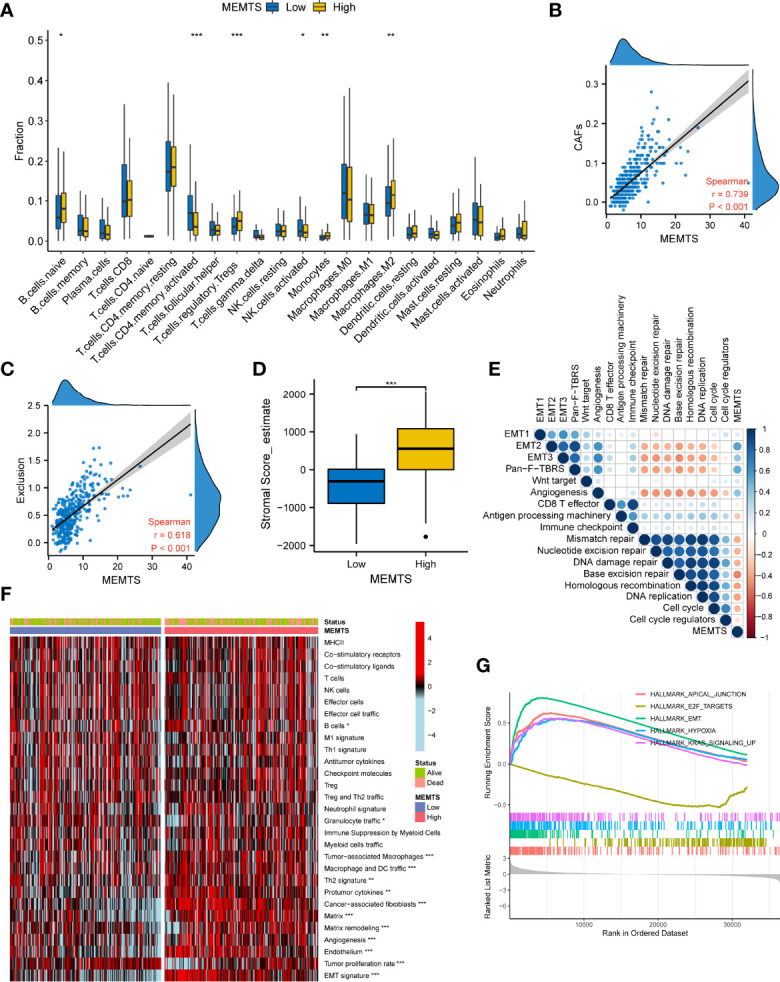
Correlation between the MEMTS and tumor microenvironment in gastric cancer (TCGA cohort). **(A)** Box plots illustrating the relationships between MEMTS subtypes and the infiltration of 22 immune cells (*P < 0.05; **P < 0.01; ***P < 0.001, Wilcoxon test). **(B)** Spearman analysis of correlation between MEMTS and CAFs (Spearman test, r=0.739, P < 0.001). **(C)** Scatter plot depicting a close correlation between MEMTS and T cell exclusion (Spearman test, r=0.618, P < 0.001). **(D)** stromal score calculated by estimate were significantly associated with MEMTS subtypes (Wilcoxon test, ***P < 0.001). **(E)** A corrplot demonstrating correlations among MEMTS and the known gene signatures in TCGA cohort by Spearman analysis. Coefficients are characterized in color and size. Negative and positive correlations are marked with orange and blue, respectively. **(F)** heatmap displaying the relationship among the MEMTS, the status and the pathways related to immunity and tumorigenesis in two subtypes (*P < 0.05; **P < 0.01; ***P < 0.001, Wilcoxon test). **(G)** Curves showing the results of gene enrichment in apical junction, E2F targets, EMT, hypoxia pathways and KRAS signaling.

Tumor microenvironment (TME) played a fundamental role in tumor progression and therapeutic response. To understand the correlation between tumor immune microenvironment and MEMTS subtypes, we used CIBERSORT algorithm to evaluate the distribution of 22 infiltrated immune cells in MEMTS-high and -low subtypes. It was observed that the MEMTS-high subtype exhibited significantly higher infiltration of immunosuppressive cells including T cell regulatory (Tregs) and M2 subtype of macrophages **(** **Figure 6A** **)**. With respect to non-immune cells, MEMTS was significantly correlated with cancer associated fibroblasts (CAFs) **(** **Figure 6B** **)**. As CAFs act as stromal cell clusters to exclude T cell infiltration and function (22), we further explored the relationship between MEMTS, stromal score, and T cell exclusion and uncovered that MEMTS was positively associated with both exclusion and stromal score in GC **(** **Figures 6C, D** **)**. To characterize the function of MEMTS, we assessed the relationship between MEMTS and the known molecular signatures **(** **Figure 6E** **)**. Of note, stroma-activated pathways such as EMT2, EMT3, Panfibroblast TGF-β response characteristics (Pan-F TBRS), and angiogenesis were found to be positively associated with MEMTS while tumor suppressive pathways were negatively related to MEMTS. The oncogenic and immunosuppressive role of MEMTS in GC was illustrated in the heatmap based on ssGSEA analyses **(** **Figure 6F** **)**. Similarly, the oncogenic pathways such as E2F targets, hypoxia, and EMT were remarkably enriched in MEMTS-high subtype **(** **Figure 6G** **)**. These results taken together reveal the relationship between MEMTS and TME, which potentially explains the predictive value of MEMTS in adjuvant therapy response in GC.

**Figure 7:**
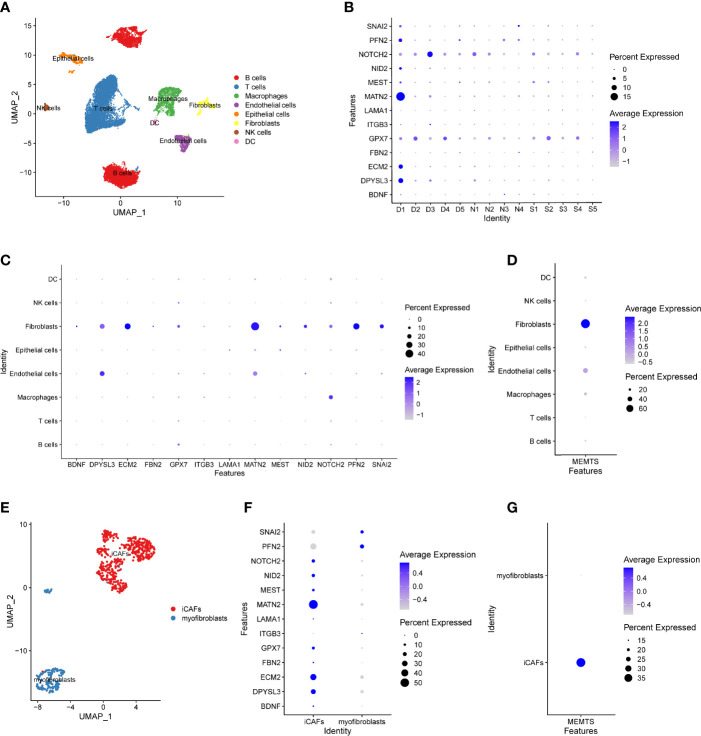
The distribution of the MEMTS in tumor microenvironment. **(A)** UMAP plot showed eight cell types from 19,765 cells. **(B)** The different features of hub genes in deep layer (D1, D2, D3, D4, D5) and superficial layer (S1, S2, S3, S4, S5) of tumor tissues and paired normal tissues (N1, N2, N3, N4). **(C)** The different expression of hub genes in eight cell clusters. **(D)** MEMTS was mainly concentrated in the fibroblasts. **(E)** Fibroblasts were classified into inflammatory CAFs and myofibroblasts on the basis of different molecular characteristics. **(F)** The different features of hub genes in inflammatory CAFs and myofibroblasts. **(G)** MEMTS was primarily manifested in inflammatory CAFs.

Recently, single-cell RNA sequencing (scRNA-seq) has emerged as a powerful technology to characterize molecular features of individual cells, which enables the highly accurate understanding of tumor microenvironment (23). To further address the role of MEMTS in TME, we analyzed GSE167297 dataset which was derived from the scRNA-seq analysis of single cells from diffuse-type GC using 10X Genomics. By using UMAP algorithm, 19,765 cells screened out by quality control were divided into eight cell clusters, each of which was annotated with cell lineage based on the cell lineage marker genes. The majority of the annotated cell clusters were immune cells including B cells, T cells, macrophages, NK cells, and dendritic cells (DC) **(** **Figure 7A** **)**. Apart from the above immune cells, stromal cells such as fibroblasts, endothelial cells, and epithelial cells were the non-negligible ingredients in single-cell atlas. The hub genes of MEMTS were detected mainly in the deep layers of GC tissues **(** **Figure 7B** **)**. Then we explored the expression of hub genes in eight cell clusters. Intriguingly, most hub genes were highly expressed in fibroblast while NOTCH2, DPYSL3, and MATN2 were also expressed at considerably high levels in endothelial cells or macrophages **(** **Figure 7C** **)**. In addition, in consistency with previous results, MEMTS was mainly enriched in the fibroblasts, followed by endothelial cells and macrophages **(** **Figure 7D** **)**. Consequently, we further explored the potential association of hub genes with fibroblasts which were classified into inflammatory CAFs (iCAFs) and myofibroblasts **(** **Figure 7E** **)**. iCAF clusters are featured with high expression of chemokines including CXCL1, CXCL14, CCL2, and interleukin 33 (IL33) while myofibroblast clusters are featured with high expression of ACTA2. A majority (11/13) of hub genes were mainly expressed in iCAFs except for SNAI2 and PFN2 **(** **Figure 7F** **)**. Notably, MEMTS was primarily manifested in iCAFs rather than myofibroblasts **(** **Figure 7G** **)**. Taken together, our analyses uncovered the relationship between MEMTS and TME, which not only indicated the significance of MEMTS in tumor immunity and metastasis but also facilitated us to better understand the predictive value of MEMTS in multiple types of adjuvant therapy in GC.

## Discussion

GC is a disease accompanied by heavy social economic burden, high incidence, and high mortality (24). Despite great advancements in treatment of GC, tumor recurrence caused by metastasis and drug resistance are still threats to patients with GC (1, 25). As one of the essential processes of tumor metastasis, EMT referred to the transdifferentiation of epithelial phenotypes into mesenchymal phenotypes. EMT enhanced the ability of tumor cells in migration and invasion, and the expression of certain genes could be used to explore the extent of EMT. In this study, we intersected the gene set associated with EMT and differentially expressed genes between primary gastric tumor and ovarian metastatic tumor. Subsequently we obtained a gene set containing 13 genes and analyzed the correlations and characteristics among them.

Increasing research has revealed the significant relationship between gene mutations and the metastasis of tumor. Tumor mutation burden (TMB) also played a crucial role in improving immunotherapy response in treatment of cancer on account of increased tumor neoantigens expression (16). It has been reported that EMT was negatively related to TMB due to the switch of MLH1 from silence to activation (26). MLH1 was responsible for gene mismatch repair and it was remarkably silenced by methylation of its promoter regions. What’s more, MSI molecular subtype of GC was characterized by higher mutation rates and hypermethylation at MLH1 promoter (27). However, it was activated to make DNA repair system intact when EMT occurred, leading to a lower mutation rate. Our results also showed that the high MEMTS subtype showed lower somatic mutation rate and TMB while the low subtype showed the opposite result. Additionally, several studies have demonstrated that patients with the MSI and EBV subtypes of GC were more sensitive to PD1 inhibitors such as pembrolizumab (28). According to our study, MEMTS was significantly lower in the EBV subtype and MSI subtype compared to the other two subtypes. These results further demonstrate that the MEMTS could be used as a robust model for stratifying patients with GC.

Tumor microenvironment (TME) had a noticeable impact on not only progression of tumor but also therapeutic response and clinical outcome. Tumor-infiltrating immune cells and carcinoma-associated fibroblasts (CAFs) within tumor stroma made the crucial contributions to the activation of EMT progression (29). CAFs in prostate cancer induced EMT *via* secretion of MMPs, which promotes the dissociation of extracellular domain of E-cadherin (30). JAK2/STAT3 pathway was activated by CAFs to promote EMT (31). Stromal cells and altered extracellular matrix also contributed to a sophisticated fiber network in favor of migration and invasion of tumor cells (32). Various molecules originated from CAFs and tumor-infiltrating immune cells such as TGF-β, FGF, EGF, HGF, and IGF1 along with Hedgehog, Notch, and Wnt signaling pathways could promote EMT (33). Apart from promotion of EMT as previously mentioned, TGFβ stimulated tumor development by motivating angiogenesis and fibroblast activation, and attenuated PD-L1 inhibitor response through exclusion of T cells, empowering tumor cells to evade antitumor immune responses (34, 35). HIF-1 downregulated the expression of E-cadherin indirectly by intensifying the expression of ZEB1, ZEB2, and TCF3 and also directly activated the TWIST1 promoter, which demonstrated that hypoxia could contribute to EMT development (36). Fully exploring the alterations of TME characteristics induced by distinct MEMTS patterns, our study showed MEMTS signatures have been investigated to be apparently related to immune infiltration and TME alteration.

Then we explored the role of MEMTS in therapeutic prediction. Aimed at improving antitumor immune response, immunotherapy has revolutionized the paradigm for tumor treatment. Immune checkpoint inhibitors are one of the most profoundly explored immunotherapies for the moment. Improvement in overall survival has been demonstrated in PD1, PDL1, and CTLA4 checkpoint inhibitor strategies compared to conventional chemoradiotherapy (3). However, success of antitumor therapy was usually limited by poor immunotherapy response and the development of drug resistance, which might result from insufficient quantity of infiltrating T cells, absence of checkpoints expression in both tumor cells and T cells, and adapted resistance to checkpoint blockade (37). As to EMT, mesenchymal subtype was widely perceived as a negative role in predicting immunotherapy response and a dominant factor of poor survival in gastric cancer, because it could facilitate immune escape through obstructing drug penetration to the core of tumors by altered TME and insufficient susceptibility to immune effector cells (38). Most recently, it has been explored that EMT simultaneously increased drug resistance through overexpressing ATP-binding cassette (ABC) transporter family and increasing cellular resistance to drug-induced apoptosis (39–41). In our research, more mutation rate in low MEMTS led to immune-checkpoint gene overexpression. Moreover, in KIM cohort, MEMTS showed higher AUC than that of MSI and EBV status. In the Hugo cohort, MEMTS had inverse correlation with PDCD1 expression and AUC of MEMTS was higher compared to PDCD1. All the above results demonstrate MEMTS is an advantageously predictive tool in precision immunotherapy for gastric cancer.

The application of MEMTS must be exercised cautiously as certain limitations of this study are noted. Firstly, the construction of MEMTS is based on the DEGs from transcriptomic analyses of paired primary GC and ovarian metastatic tumors. Therefore, the validity of MEMTS in GC patients with other types of metastases are yet to be investigated even though we tried to address this issue by not only intersecting DEGs with EMT-related gene sets from publicly available databases but also by assessing its predictive value in multiple independent cohorts. Moreover, considering that these cohorts are merely retrospective, further prospective clinical trials are required to validate our findings, especially the predictive role of MEMTS in terms of prognosis and response to various therapeutic types.

To sum up, we constructed an EMT gene set score to stratify GC patients in various cohorts and explored the underlying mechanisms leading to different characteristics between high and low MEMTS samples, which improved our understanding of EMT progression in GC. The results show that the MEMTS could be used to stratify patients and identify those who would benefit more from adjuvant therapy and immunotherapy and to detect brand new strategies and targets for cancer treatment.

## Data Availability Statement

The datasets presented in this study can be found in online repositories. The names of the repository/repositories and accession number(s) can be found below: https://www.ncbi.nlm.nih.gov/geo/, GSE191139.

## Ethics Statement

The studies involving human participants were reviewed and approved by Clinical Research Ethics Committee of Fudan University Shanghai Cancer Center. The patients/participants provided their written informed consent to participate in this study.

## Author Contributions

XL and JG conceived and designed this study. JS and SH performed the experiments. JS and RW analyzed the data and drafted the manuscript. All authors contributed to the article and approved the submitted version.

## Funding

This study was sponsored the Natural Science Foundation of Shanghai (21ZR1414600), and the Shanghai Pujiang Program (2019PJD007). The funders had no role in study design, data collection and analysis, decision to publish, or preparation of manuscript.

## Conflict of Interest

The authors declare that the research was conducted in the absence of any commercial or financial relationships that could be construed as a potential conflict of interest.

## Publisher’s Note

All claims expressed in this article are solely those of the authors and do not necessarily represent those of their affiliated organizations, or those of the publisher, the editors and the reviewers. Any product that may be evaluated in this article, or claim that may be made by its manufacturer, is not guaranteed or endorsed by the publisher.

## Supplementary Material

Supplementary Figure 1 The correlation between MEMTS and the expression of immune checkpoints. **(A)** The correlation between MEMTS and the expression of PD-L1. **(B)** The correlation between MEMTS and the expression of PD-1.
